# The effect of local Suicide Prevention Action Networks (SUPRANET) on stigma, taboo and attitudes towards professional help-seeking: an exposure–response analysis

**DOI:** 10.1007/s00127-021-02078-w

**Published:** 2021-05-01

**Authors:** Margot C. A. van der Burgt, Aartjan T. F. Beekman, Adriaan W. Hoogendoorn, Guus Berkelmans, Gerdien Franx, Renske Gilissen

**Affiliations:** 1 113 Suicide Prevention, Amsterdam, The Netherlands; 2 Department of Psychiatry, Amsterdam Public Health Research Institute, Amsterdam University Medical Centers, Amsterdam, The Netherlands; 3 GGZ inGeest Specialized Mental Health Care, Amsterdam, The Netherlands; 4 Centrum Wiskunde & Informatica (CWI), Amsterdam, The Netherlands

**Keywords:** Suicide prevention, SUPRANET, Multilevel intervention, Attitudinal change, Help-seeking behaviour

## Abstract

**Purpose:**

In 2017, the European Alliance against Depression (EAAD) programme was introduced in the Netherlands through the creation of six local Suicide Prevention Action Networks (SUPRANET Community). This programme consists of interventions on four levels: (1) a public awareness campaign, (2) training local gatekeepers, (3) targeting high-risk persons in the community and (4) training of primary care professionals. This study aims to gain insight into the effectiveness of the SUPRANET programme on attitudinal changes in the general public by studying the exposure–response relationship.

**Methods:**

A repeated cross-sectional design, using general population surveys to measure key variables over time. The surveys were conducted in the six intervention regions (*N* = 2586) and in the Netherlands as a whole as a control region (*N* = 4187) and include questions on socio-demographic variables, brand awareness of the Dutch helpline, perceived taboo on suicide, attitudes towards depression and help-seeking. To examine the exposure–response relationship, regions were differentiated into 3 groups: low, medium and high exposure of the SUPRANET programme.

**Results:**

The results revealed that respondents in the intervention regions considered professional help to be more valuable and were more likely to be familiar with the Dutch helpline than respondents in the control region. In the exposure–response analyses, the grading of effects was too small to reach statistical significance.

**Conclusion:**

Our study provides the first evidence for the effectiveness of the SUPRANET Community programme on creating attitudinal change in the general public.

## Introduction

In the Netherlands, 1811 people died by suicide in 2019 [1], more than two and a half times the number of fatal road casualties of that same year (661) [2]. Dutch suicide rates appear to have stabilised since 2013 with around 11 suicides per 100,000 inhabitants, of whom more than two-thirds (68%) are men [3]. Almost 40% of Dutch citizens who died by suicide were in specialised mental health care treatment at the time of their death, meaning that the majority did not find their way to specialised care [4].

The World Health Organisation (WHO) recognises suicide as preventable and argues that it should be high on the global public health agenda. It proposes that it is essential to use comprehensive multisectoral strategies with community-level approaches that take social, psychological and cultural impact into account. As suicide is surrounded by stigma, shame and misunderstanding, community engagement is vital. Communities can reduce risk and strengthen protective factors by providing social support and follow-up care, raising awareness, fighting stigma and supporting those bereaved by suicide [5]. Raising awareness and fighting stigmas on help-seeking behaviour is crucial as research shows that stigmas related to mental health disorders and -services are an important reason for insufficient help-seeking [6–8]. For this reason, focussing on attitudinal factors is a key aspect of suicide prevention policies.

In 2001, a four-level intervention programme was implemented in the German city of Nuremberg, called The Nuremberg Alliance against Depression (NAD). The NAD was a high-intensity, 2-year action programme against depression and suicidality. Evaluation of the programme showed a significant reduction in the number of suicidal acts [9, 10]. Since then, this four-level intervention concept has been adopted by more than 70 regions in Germany within the German Alliance against Depression and has led in 2004 to a European-wide collaboration funded by the European Commission; the European Alliance against Depression (EAAD) [11]. The research project OSPI-Europe (Optimising Suicide Prevention Programmes and their Implementation in Europe) has evaluated the intervention in four European cities in Germany, Hungary, Portugal and Ireland. Aggregated data demonstrated no significant effect of the intervention on suicidal behaviour. The previously observed preventive effects on suicidal behaviour in the Nuremberg programme were only replicated in Portugal [12]. In contrast, Hofstra and colleagues conducted a systematic review and meta-analysis and found that suicide prevention interventions are effective in preventing both completed and attempted suicides, with a significant effect of the number of levels in the intervention on effect size [13].

This paper provides a report of a test of the effects of an intervention based on the shared multilevel approach of the EAAD model, namely: the Suicide Prevention Action Network (SUPRANET) Community programme [14]. This programme was introduced in six intervention regions in the Netherlands in January 2017, as part of the Dutch National Agenda for Suicide Prevention. Although both interventions consider suicidality to be an important outcome measure, there is a significant difference in communication strategy. While EAAD's public awareness campaign focuses mainly on depression, the SUPRANET campaign addresses suicide directly [14, 15]. The SUPRANET programme is coordinated by 113 Suicide Prevention, the suicide prevention helpline and expertise centre in the Netherlands.

A companion paper on the SUPRANET public awareness campaign, showed that the campaign was predominantly visible among the younger generation (< 25 years). The respondents who indicated having seen the public awareness campaign showed more openness towards seeking professional help and were considerably more likely to be familiar with the Dutch suicide prevention helpline. In contrast to expectations, campaign awareness also seemed to relate to a higher perceived taboo on suicide and a lower estimation of the value of professional help [16].

Because of the interactions between components and the synergistic effects that might arise within a multilevel prevention programme, it is not possible to determine the reach and contribution of each component in the totality of this intervention [17, 18]. Given this, the aim of our quasi-experimental study was to gain insight into the impact of the Suicide Prevention Action Networks on attitudinal changes in the general public by studying the exposure–response relationship. This is possible because the level of implementation of the intervention varied across the six intervention regions. We hypothesise that the attitudinal change and brand awareness of the Dutch helpline among the general public was greater in the intervention regions as compared to the control region and the highest in the region with the most exposure to the SUPRANET Community intervention. The findings of this study will be used to facilitate the nationwide implementation of this programme and will add to a growing body of research on the effectiveness of multilevel prevention programmes.

## Methods

### The SUPRANET community programme

**Fig. 1:**
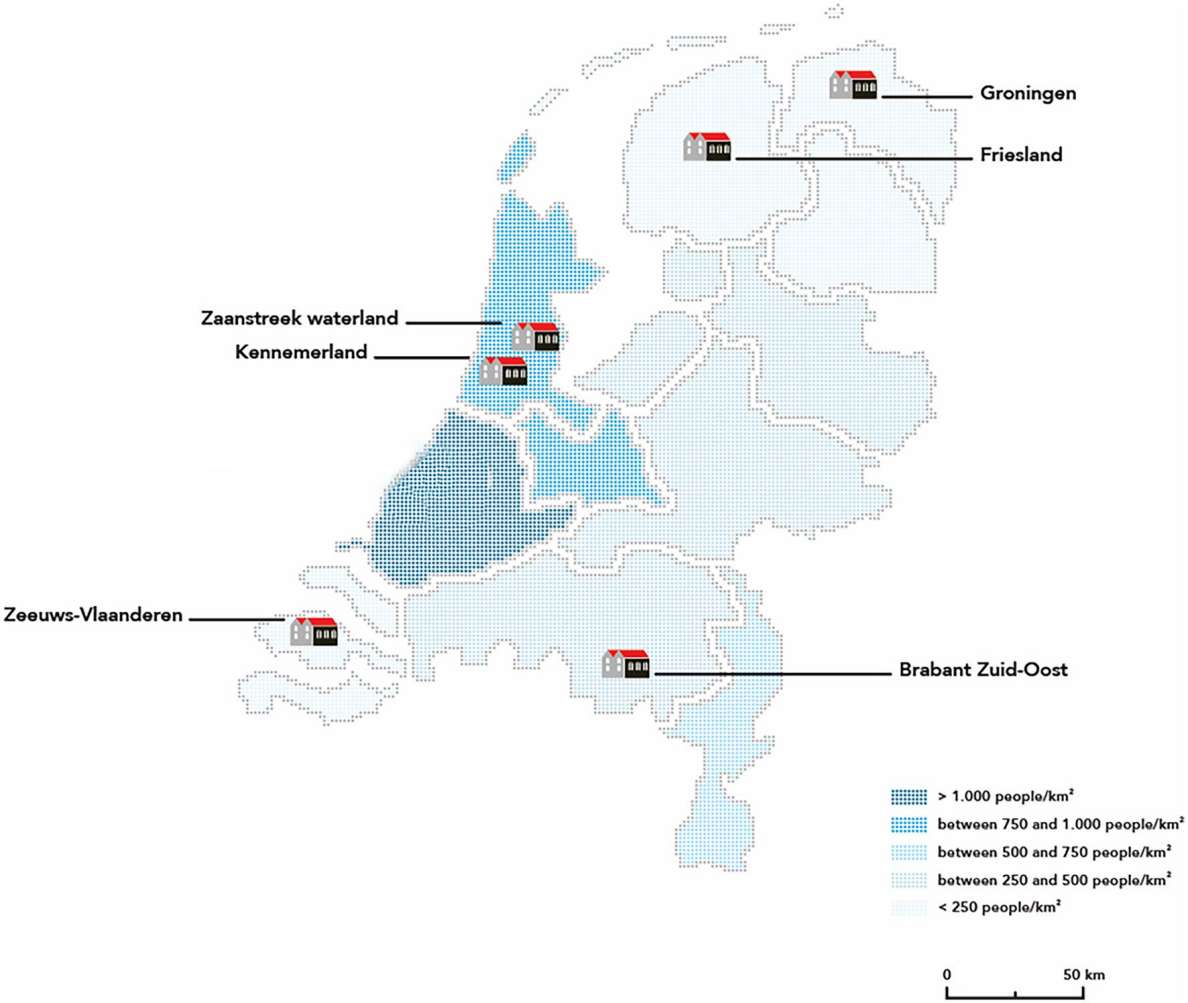
The six SUPRANET communities. Colouring represents the population density of the region

In 2017, the SUPRANET Community programme was introduced in the Netherlands. SUPRANET Community is a 2-year multicomponent and multisetting suicide prevention programme. It was modelled on EAAD and consists of four levels: (1) increasing the awareness of suicide by local public awareness campaigns; (2) training local gatekeepers; (3) targeting high-risk persons in the community; and (4) the training and support of professionals in primary care settings. The implementation of the programme started in six intervention regions (see Fig. 1). In such a community, a network of local multidisciplinary teams and organisations shared ownership and responsibility for the prevention of suicide within a geographical region. More information on the selection process of the intervention regions can be found in the study protocol by Gilissen et al. [14].

#### Level 1: local public awareness campaigns

Local public awareness campaigns were based on a national campaign launched by 113 Suicide Prevention. The campaign called *De vraag van je leven*, which can be translated as “The question of your life”, was aimed at the general public, as well as healthcare providers, general practitioners and other professionals. The campaign carried the message that asking one question can make a difference and intended to contribute to breaking the taboo on talking about suicide. The campaign aimed to educate the public in recognising the signs of suicidal thoughts, giving practical tips and guidelines on how to manage suicidality and bring to the attention that one can turn to 113 Suicide Prevention for help. The national public awareness campaign had five different “waves” of two or three weeks and started in February 2017 and ended in November 2018. More detailed information about the national and regional public awareness campaigns can be found in Van der Burgt et al. [16].

#### Level 2: training local gatekeepers

The fact that the majority of people who died by suicide did not find their way to specialised care, illustrates the importance of recognising and assisting people with suicidal ideation within community settings. In 2008, the gatekeeper training was tailored for implementation in the Netherlands [19]. Since then, many professionals in various employment sectors, such as teachers, police officers, railway employees and healthcare professionals, have completed the training. The Dutch gatekeeper training has shown to be effective in improving participant’s knowledge on suicide and addressing suicidality and in their self-confidence to conduct a dialogue on suicide and suicidal thoughts [20].

#### Level 3: targeting high-risk persons in the community

The SUPRANET regions were encouraged to actively approach specific risk groups, who are known to be especially vulnerable for developing suicidal thoughts. In some regions this included an intervention for people within the LGBT + community, other regions preferred to target people on disability or welfare, youth, students, farmers or middle-aged people (especially men). Examples of interventions include providing information about suicide prevention at schools and establishing contacts with organizations involved or working with high-risk groups.

#### Level 4: the support of professionals in primary care settings

The final component of the SUPRANET Community intervention includes the support of primary care professionals (PCPs) to stimulate the signalling and exploration of suicidal ideation and concrete suicide plans among their patients. In the SUPRANET regions, a suicide prevention training was offered to primary care professionals. This training was based upon the multidisciplinary guideline for diagnosis and treatment of suicidal behaviour [21]. In addition to the training, materials were offered to the PCPs. These materials included a checklist to use during consultations, flyers and posters aimed at patients to encourage talking about suicidal thoughts and modules on suicide and medication to inform about which medication might induce or reduce suicidality and which medication is often used for suicide attempts [22].

### The exposure–response measure

The implementation of the SUPRANET Community programme varied between the six intervention regions, with most of them missing components of the EAAD model. There was only one region, in the north of the Netherlands, where the intervention was implemented as intended in the studied timeframe. In this region, general practitioners and gatekeepers were trained, the public awareness campaign was widely spread, with posters placed in various public places (like pharmacies, supermarkets, churches, sports clubs and inside buses). Advertorials were used, information meetings were organised and there was a collaboration with local broadcasters for radio- and TV spots and interviews in the local language. High-risk groups were, amongst others, targeted by a photo exposition aimed at LGBT+ youth, participation in a local canal pride and collaborations with the educational and agricultural sector. The national public awareness campaign was visible across the country but only the intervention regions were part of the multilevel and multicomponent intervention. For this reason, the Netherlands as a whole was categorised as “low exposure”. The one region that had the ultimate implementation of all four levels was categorised as “high exposure”. The remaining five intervention regions, that managed to implement two or three levels, were labelled as “medium exposure”.

### Study design and procedure

**Fig. 2:**
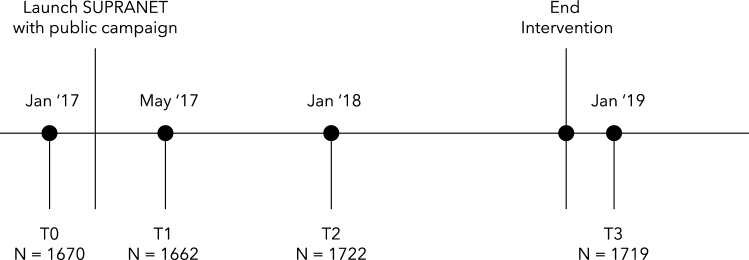
Timeline of the four measurements

This study has a repeated cross-sectional design and uses general population surveys to measure key variables over time. The surveys were conducted in the six intervention regions and in the Netherlands as a whole as a control region. Respondents were recruited through Survey Sampling International (now known as Dynata), which maintains a demographically diverse online panel of people who have registered to participate in selected surveys. When members of the panel log onto Dynata’s website, they are randomly directed (with the use of a survey router system) to available surveys based on their demographic characteristics. The panel members also receive a modest reward [23]. Due to this router system, it was not possible to collect response rates. Although Dynata made several efforts to ensure that the samples in this study represent different age groups, educational levels, genders, household situations and occupational statuses, it should be considered as a convenience sample. The data consists of four measurements, timed to be able to capture the effect of the intervention as best as possible. The baseline measurement was conducted before SUPRANET Community’s start in January 2017 (T0, *N* = 1670). The second measurement took place just after the first public awareness campaign wave in May 2017 (T1, *N* = 1662). The third and fourth measurements took place one year (T2, *N* = 1722) and  two years after the start of SUPRANET Community (T3, *N* = 1719), respectively. Respondents in longitudinal studies may become conditioned to the study in which they are participating; they may remember and repeat their previous answers, or become sensitised to the research topic [24]. This is why each measurement contains a new representative sample of adults (aged 18 +) from the Dutch general population; 1000 for the control region and approximately 100 per intervention region. Figure 2 illustrates a timeline of those four measurements with the number of respondents for each measurement.

### Survey instrument

The same digital survey was used for each measurement. This survey gathered information on sociodemographic information (including gender, age, region, educational level and household situation), brand awareness of the Dutch helpline and perceived taboo on suicide. Moreover, the Dutch Depression Stigma Scale (DSS) and the Attitude towards Seeking Professional Psychological Help Short Form (ATSPPH-SF) were included. The Dutch DSS measures the stigma associated with depression and consists of two subscales: the Personal Stigma Scale and the Perceived Stigma Scale. Both subscales consist of nine items about depression. Responses to the items are measured on a five-point Likert scale ranging from “strongly disagree” to “strongly agree” [25]. ATSPPH-SF also contains two subscales: “Openness to Seek Treatment for Emotional Problems” and “Value and Need in Seeking Treatment”. Both subscales consist of five items assessed on a four-point Likert scale ranging from “disagree” to “agree” [26]. More detailed information about the survey instrument can be found in Van der Burgt et al.[16].

### Statistical analysis

All statistical analyses were conducted using IBM SPSS Statistics version 25.0 and the “stats” package in R version 3.5.1. To investigate the overall impact of the intervention, the six intervention regions have been as compared to the control region using multivariable linear regression models to predict respondents’ scores on the five continuous outcomes: perceived taboo, the two subscales of the DSS and the ATSPPH-SF. Multiple logistic regression analysis was used for the binary outcome; brand awareness. Time, age, gender and educational level were expected to have an effect on the key variables and were, therefore, included as confounders. In order to gain insight into the differences between the intervention regions and the control region over time, these models were extended with interaction variables between “time” and “intervention region”. Additionally, to investigate the exposure–response relationship, the effect of “high exposure” (i.e. living in a high exposure region) and “medium exposure” as compared to control category “low exposure”, these models were extended by adding “level of exposure region” by “time” interaction terms as predictors. To measure the internal consistency of the ATSPPH-SF and DSS subscales in this sample, the Cronbach’s alpha value of each subscale was calculated.

## Results

Between January 2017 and January 2019, four surveys have been carried out. Table 1 displays the respondents’ characteristics of those four measurements. These characteristics were comparable regarding gender, educational level and household situation. Only age (*χ*2 (15) = 83.18, *p* < 0.05) and occupational status (*χ*2 (21) = 68.40, *p* < 0.05) of the respondents were significantly different between the four measurements. We could not come up with an explanation of why these differences occurred and attributed these differences to chance. In this sample, the Cronbach’s alpha value of the two ATSPPH-SF subscales ‘Openness to Seek Treatment for Emotional Problems’ and ‘Value and Need in Seeking Treatment’ were 0.78 and 0.67 respectively. The alpha values of the DSS subscales were 0.84 for personal stigma and 0.85 for perceived stigma.

**Table 1:** Respondents’ characteristics (in %)

	T0	T1	T2	T3
	(*N* = 1670)	(*N* = 1662)	(*N* = 1722)	(*N* = 1719)
Gender				
Female	50.4	51.6	53.6	51.3
Male	49.6	48.4	46.4	48.7
Age group				
< 25 years	12.2	12.5	14.5	12.7
25–34 years	11.4	10.8	12.1	12.3
35–44 years	13.2	11.7	14.5	12.4
45–54 years	23.5	24.5	18.7	22.3
55–64 years	26.0	23.3	21.4	18.4
> 65 years	13.6	17.3	18.8	21.8
Educational level				
Low	31.3	31.2	30.3	30.4
Medium	41.0	40.4	40.7	41.6
High	27.7	28.4	29.0	28.0
Household situation				
Married/cohabitating, with children	41.8	38.7	39.5	40.5
Married/cohabitating, without children	16.4	17.2	17.8	16.3
Single, with children	7.8	7.3	7.4	7.6
Single, without children	21.7	25.3	20.9	23.3
Community housing, student house	2.0	2.5	2.3	2.0
Living at parents or family	8.6	7.5	10.0	8.8
Other	1.7	1.6	2.0	1.5
Occupational status				
Employed	37.1	35.4	37.0	39.6
Entrepreneur	6.1	7.5	7.0	5.9
Working for the government	3.7	3.2	3.1	3.6
Incapacitated/unemployed/ looking for a job/social benefits for health reasons	13.7	15.3	12.1	12.0
Unemployed/looking for a job/social benefits due to other reasons	5.3	5.5	3.3	2.9
Retired	15.4	16.5	18.2	20.7
Student	8.1	8.1	9.2	7.5
Housekeeping /other	10.7	8.5	10.0	7.9

Notice that the data have a hierarchical structure, as the respondents were clustered within regions. We, therefore, initially tried fitting multilevel models to take this clustering into account. However, multilevel analyses in *R* indicated a very small intraclass correlation, most likely due to the homogeneity of the Netherlands as a small country. Likewise, adding the regions as a second level did not significantly improve the models.

Table 2 presents the multiple regression models predicting respondents’ scores on the six key variables; brand awareness of 113 Suicide Prevention, perceived taboo on suicide, the two DSS subscales (perceived and personal stigma towards depression) and the two ATSPPH-SF subscales (Openness to Seek Treatment for Emotional Problems and Value and Need in Seeking Treatment). Taking a focus on the effect of the intervention, the results show that respondents in the intervention regions scored significantly better on four out of six key variables. When controlled for time, age, educational level and gender, respondents in the intervention regions were considerably more likely to be familiar with the Dutch helpline (OR = 1.26, *p* < 0.001) and scored 0.38 point higher on the Value and Need in Seeking Treatment subscale, that ranges from 0 to 15 (*β* = 0.38, *p* < 0.001) as compared to the respondents in the control region. Moreover, respondents in the intervention regions showed less personal (*β* = − 1.01, *p* < 0.001) and perceived stigma (*β* = − 0.73, *p* < 0.001) towards depression than respondents in the control region. The results indicated no significant difference in the perceived taboo on suicide and the scores on the Openness to Seek Treatment for Emotional Problems subscale.

**Table 2:** Overview of regression models predicting the scores on six key variables

	Brand awareness			Perceived taboo		Perceived stigma subscale		Personal stigma subscale		Openness subscale		Value subscale	
	ln(OR)	SE	OR (95% CI)	B	SE	B	SE	B	SE	B	SE	B	SE
Constant	− 1.28***	0.12	0.28	5.52***	0.11	17.52***	0.31	15.83***	0.30	7.93***	0.15	7.56***	0.14
Measurement (ref: T0)													
T1	1.37***	0.09	3.92 (3.31–4.65)	0.05	0.07	0.74***	0.22	0.06	0.21	0.13	0.11	− 0.01	0.10
T2	1.40***	0.09	4.06 (3.42–4.82)	− 0.16*	0.07	0.16	0.22	0.20	0.21	0.21	0.11	− 0.09	0.10
T3	2.25***	0.09	9.48 (7.99–11.25)	0.11	0.07	0.61**	0.22	-0.01	0.21	0.23*	0.11	− 0.18	0.10
Age group (ref: < 25 years)													
25–34 years	− 0.24*	0.11	0.78 (0.63–0.97)	0.49***	0.11	1.09***	0.31	0.42	0.30	0.51***	0.15	0.31*	0.14
35–44 years	− 0.79***	0.11	0.45 (0.37–0.56)	0.65***	0.10	1.18***	0.30	0.49	0.30	0.46**	0.15	0.59***	0.13
45–54 years	− 0.89***	0.10	0.41 (0.34–0.50)	0.82***	0.09	− 0.04	0.27	− 0.95***	0.27	0.67***	0.13	0.85***	0.12
55–64 years	− 0.87***	0.10	0.42 (0.35–0.51)	0.99***	0.09	− 0.93***	0.27	− 1.46***	0.27	0.93***	0.13	0.98***	0.12
> 65 years	− 0.98***	0.10	0.38 (0.31–0.46)	0.76***	0.10	− 2.43***	0.29	− 1.07***	0.29	1.03***	0.14	0.83***	0.13
Education level (ref: low)													
Middle	0.13	0.07	1.14 (1.00–1.30)	0.21***	0.06	0.07	0.19	− 0.95***	0.18	0.15	0.09	0.32***	0.08
High	0.36***	0.07	1.44 (1.24–1.67)	0.36***	0.07	0.20	0.21	− 2.13***	0.21	0.49***	0.10	0.62***	0.09
Gender (ref: male)													
Female	− 0.13*	0.05	0.88 (0.79–0.98)	0.27***	0.05	− 0.94***	0.16	− 2.88***	0.15	0.42***	0.08	0.77***	0.07
Intervention region (ref: no)													
Yes	0.23***	0.06	1.26 (1.12–1.41)	0.07	0.06	− 0.73***	0.16	− 1.01***	0.16	0.16	0.08	0.38***	0.07

*CI* confidence interval

*p < 0.050; **p < 0.010; ***p < 0.001

A more in-depth analysis (see Table 3) on the differences between the intervention regions and the control region over time, shows an increasing trend in the proportion of respondents who are familiar with 113 Suicide Prevention in both the control region and the intervention regions, with a significantly larger proportion in the intervention regions at T3 (OR = 1.43, *p* < 0.050). The difference between the intervention regions and the control region on the Value and Need in Seeking Treatment subscale seems to be driven by a significantly higher score in the intervention regions at T2 (*β* = 0.43, *p* < 0.050). The initially found difference between the intervention regions and the control region on personal stigma towards depression can be explained by a difference at baseline; the intervention regions already scored significantly lower on personal stigma regarding depression (*β* = − 0.68, *p* < 0.050) before the start of the intervention at T0. The earlier found difference in perceived stigma towards depression also disappears when we take the different measurements into account. The interaction variables reveal fluctuations in the intervention regions regarding perceived stigma towards depression, with a lower perceived stigma in the intervention regions at T1 and T3 but higher at T2.

**Table 3:** Overview of regression models predicting the scores on six key variables including interaction variables

	Brand awareness			Perceived taboo		Perceived stigma subscale		Personal stigma subscale		Openness subscale		Value subscale	
	ln(OR)	SE	OR (95% CI)	B	SE	B	SE	B	SE	B	SE	B	SE
Constant	− 1.22***	0.12	0.30	5.49***	0.11	17.47***	0.33	15.71***	0.32	7.94***	0.16	7.62***	0.14
Measurement (ref: T0)													
T1	1.33***	0.11	3.78 (3.05–4.69)	0.14	0.09	0.79**	0.27	0.02	0.27	0.12	0.13	− 0.09	0.12
T2	1.34***	0.11	3.83 (3.09–4.76)	− 0.20*	0.09	0.10	0.27	0.42	0.27	0.17	0.13	− 0.25*	0.12
T3	2.12***	0.11	8.30 (6.70–10.28)	0.18	0.09	0.83**	0.27	0.30	0.27	0.21	0.13	− 0.22	0.12
Age group (ref: < 25 years)													
25–34 years	− 0.25*	0.11	0.78 (0.63–0.97)	0.49***	0.11	1.10***	0.31	0.43	0.30	0.51***	0.15	0.32*	0.14
35–44 years	− 0.79***	0.11	0.45 (0.37–0.56)	0.65***	0.10	1.19***	0.30	0.48	0.30	0.47**	0.15	0.59***	0.13
45–54 years	− 0.88***	0.10	0.41 (0.34–0.50)	0.82***	0.09	− 0.05	0.27	− 0.96***	0.27	0.67***	0.13	0.85***	0.12
55–64 years	− 0.87***	0.10	0.42 (0.35–0.51)	0.99***	0.09	− 0.93***	0.27	− 1.46***	0.27	0.93***	0.13	0.98***	0.12
> 65 years	− 0.97***	0.10	0.38 (0.31–0.46)	0.76***	0.10	− 2.43***	0.29	− 1.08***	0.29	1.03***	0.14	0.84***	0.13
Education level (ref: low)													
Middle	0.13	0.07	1.14 (1.00–1.30)	0.21***	0.06	0.08	0.19	− 0.95***	0.18	0.15	0.09	0.33***	0.08
High	0.36***	0.08	1.44 (1.24–1.67)	0.36***	0.07	0.21	0.21	− 2.13***	0.21	0.49***	0.10	0.62***	0.09
Gender (ref: male)													
Female	− 0.13*	0.05	0.88 (0.79–0.98)	0.27***	0.05	− 0.94***	0.16	− 2.88***	0.15	0.42***	0.08	0.77***	0.09
Intervention region (ref: no)													
Yes	0.06	0.14	1.06 (0.80–1.41)	0.16	0.11	− 0.60	0.32	− 0.68*	0.31	0.11	0.16	0.20	0.14
Interaction effects													
T1 * IR	0.09	0.18	1.10 (0.77–1.56)	− 0.23	0.15	− 0.11	0.45	0.09	0.44	0.02	0.22	0.21	0.20
T2 * IR	0.15	0.18	1.16 (0.81–1.65)	0.11	0.15	0.19	0.46	− 0.61	0.45	0.10	0.22	0.43*	0.20
T3 * IR	0.36*	0.18	1.43 (1.01–2.03)	− 0.20	0.15	− 0.59	0.45	− 0.82	0.44	0.05	0.22	0.11	0.20

*IR* intervention region, *CI* confidence interval

*p < 0.050; **p < 0.010; ***p < 0.001

The exposure–response relationship is analysed by comparing the high exposure region with the other five interventions regions (medium exposure) and the control region (low exposure). Table 4 presents the regression models for the six key variables. The main effect of the exposure variable displays the difference between the low, medium and high exposure regions at baseline. The interaction variables show the scores on the key variables in the medium and high exposure regions over time. For example, we see that the medium exposure regions scored significantly lower at baseline on perceived stigma than the low exposure regions (*β* = − 0.70, *p* < 0.050). When looking at the interaction variables for “perceived stigma”, we see a decreasing trend in the high exposure region over time but differences are too little to be statistically significant. Overall, the results of the exposure–response analyses show that although the high exposure region seems to score better on multiple key variables, differences were too little to show significant effects. We, therefore, have to conclude that we found no evidence for an exposure–response relationship.

**Table 4:** Overview of exposure–response regression models including interaction variables

	Brand awareness			Perceived taboo		Perceived stigma subscale		Personal stigma subscale		Openness subscale		Value subscale	
	ln(OR)	SE	OR (95% CI)	B	SE	B	SE	B	SE	B	SE	B	SE
Constant	− 1.22***	0.12	0.30	5.49***	0.11	17.47***	0.33	15.71***	0.32	7.94***	0.16	7.62***	0.14
Measurement (ref: T0)													
T1	1.33***	0.11	3.78 (3.05–4.69)	0.14	0.09	0.79**	0.27	0.02	0.27	0.12	0.13	− 0.09	0.12
T2	1.34***	0.11	3.83 (3.09–4.75)	− 0.20*	0.09	0.10	0.27	0.42	0.27	0.17	0.13	− 0.25*	0.12
T3	2.12***	0.11	8.30 (6.69–10.28)	0.18	0.09	0.83**	0.27	0.30	0.27	0.21	0.13	− 0.22	0.12
Age group (ref: < 25 years)													
25–34 years	− 0.25*	0.11	0.78 (0.63–0.97)	0.49***	0.11	1.09***	0.31	0.43	0.30	0.51***	0.15	0.32*	0.14
35–44 years	− 0.79***	0.11	0.45 (0.37–0.56)	0.64***	0.10	1.18***	0.30	0.48	0.30	0.47**	0.15	0.60***	0.13
45–54 years	− 0.88***	0.10	0.41 (0.34–0.50)	0.82***	0.09	− 0.05	0.27	− 0.97***	0.27	0.68***	0.13	0.85***	0.12
55–64 years	− 0.87***	0.10	0.42 (0.35–0.51)	0.99***	0.09	− 0.93***	0.27	− 1.46***	0.27	0.94***	0.13	0.98***	0.12
> 65 years	− 0.97***	0.10	0.38 (0.31–0.46)	0.75***	0.10	− 2.45***	0.29	− 1.09***	0.29	1.04***	0.14	0.84***	0.13
Education level (ref: low)													
Middle	0.13	0.07	1.14 (1.00–1.30)	0.21***	0.06	0.08	0.19	− 0.94***	0.18	0.16	0.09	0.32***	0.08
High	0.36***	0.08	1.44 (1.24–1.67)	0.36***	0.07	0.21	0.21	− 2.13***	0.21	0.49***	0.10	0.62***	0.09
Gender (ref: male)													
Female	− 0.13*	0.05	0.88 (0.79–0.98)	0.27***	0.05	-0.93***	0.16	− 2.88***	0.15	0.42***	0.08	0.77***	0.07
Exposure (ref: low)													
Medium	0.01	0.16	1.01 (0.75–1.37)	0.10	0.11	− 0.70*	0.34	− 0.68*	0.33	0.14	0.17	0.20	0.15
High	0.29	0.27	1.34 (0.79–2.27)	0.44*	0.22	− 0.12	0.64	− 0.67	0.63	0.01	0.31	0.16	0.28
Interaction effects													
T1*Medium	0.16	0.19	1.17 (0.81–1.71)	− 0.17	0.16	0.05	0.48	0.17	0.47	-0.05	0.23	0.20	0.21
T2*Medium	0.24	0.19	1.27 (0.87–1.85)	0.20	0.16	0.51	0.48	− 0.40	0.47	0.10	0.24	0.39	0.21
T3*Medium	0.34	0.19	1.40 (0.96–2.04)	− 0.14	0.16	− 0.38	0.48	− 0.87	0.47	0.00	0.23	0.09	0.21
T1*High	− 0.21	0.34	0.81 (0.41–1.59)	− 0.49	0.31	− 0.89	0.90	− 0.33	0.88	0.37	0.44	0.23	0.40
T2*High	− 0.29	0.35	0.75 (0.38–1.48)	− 0.28	0.31	− 1.39	0.93	− 1.65	0.91	0.12	0.45	0.63	0.41
T3*High	0.45	0.35	1.57 (0.79–3.10)	− 0.49	0.30	− 1.52	0.87	− 0.61	0.85	0.25	0.43	0.22	0.38

*CI* confidence interval. Exposure, *Low* control region, only public awareness campaign. *Medium* five regions that implemented 2 or 3 levels. *High* region that implemented all four levels

*p < 0.050; **p < 0.010; ***p < 0.001

When looking at the different predictors of the key variables we see differences between genders, age groups and educational levels. On average, women considered professional help to be more valuable (*β* = 0.77, *p* < 0.001), reported more openness towards seeking professional help (*β* = 0.42, *p* < 0.001) and reported less personal stigma (*β* = − 2.88, *p* < 0.001) and perceived stigma (*β* = − 0.94, *p* < 0.001) towards depression than men. Women were also less likely to be familiar with the Dutch helpline (OR = 0.88, *p* < 0.050) and scored higher on the perceived taboo on suicide (*β* = 0.27, *p* < 0.001). High educated people considered professional help to be more valuable (*β* = 0.62, *p* < 0.001) and reported more openness towards seeking professional help (*β* = 0.49, *p* < 0.001) than low educated respondents. High educated respondents also reported less personal stigma towards depression (*β* = − 2.13, *p* < 0.001), had more chance to be familiar with the Dutch helpline (OR = 1.44, *p* < 0.001) and scored a little higher on the perceived taboo on suicide (*β* = 0.36, *p* < 0.001) than low educated respondents.

## Discussion

The main goal of this study was to test the effectiveness of the SUPRANET Community programme in achieving attitudinal change in the general public. Given the systematic variation in the level of implementation and thereby exposure to the intervention in the regions of the Netherlands participating, we were able to test for the exposure–response relationships. We hypothesised that the attitudinal change and brand awareness of the Dutch helpline among the general public was greater in the intervention regions as compared to the control region and the highest in the high exposure region. Multiple (logistic) regression analyses revealed that respondents in the intervention regions were considerably more likely to be familiar with the Dutch helpline 113 Suicide Prevention, considered professional help to be more valuable and showed less personal and perceived stigma towards depression than respondents in the control region. Overall, four of the six outcomes that were used to test the effectiveness of the programme showed significant results.

A more in-depth analysis of the differences between the intervention regions and the control region over time shows an increasing trend in the proportion of respondents who are familiar with 113 Suicide Prevention in both the control region and the intervention regions, with a significantly larger proportion in the intervention regions at T3. The previously found difference between the intervention regions and the control region regarding the Value and Need in Seeking Treatment subscale was driven by a significantly higher score in the intervention regions at T2. The initially found difference in personal stigma towards depression can be partially explained by a difference at baseline; the intervention regions already scored significantly lower on personal stigma regarding depression before the start of the intervention. And also, the difference in perceived stigma disappears when looking at the different measurements, due to the fluctuations between T1 and T3. We could not come up with an explanation for the difference in personal stigma towards depression at baseline, or the fluctuations in perceived stigma, but the fact that these results came to light in our follow-up analysis, underlines the importance of longitudinal studies. At the higher end of the exposure, the exposure–response analyses showed that, although the high exposure region seems to score better on multiple key variables, differences were too little to yield statistically significant results.

Strengths of this study are the repeated cross-sectional design, with relatively large sample sizes, the use of internationally validated instruments and the exposure–response analyses. However, our study has some limitations that should be taken into consideration when interpreting its findings. First, a randomised controlled trial would have been a preferred design. However, for many reasons, this was not feasible. Testing for effects of systematic variation in the exposure to the intervention, utilizing a longitudinal design with multiple observations and controlling for confounders were three strategies we employed in order to test the effect of the intervention. Although this limits the causal inference, it is the best we can do given the context and the type of intervention under study. Second, self-report can lead to desirability bias, especially when measuring attitudes and stigmas. But the use of an anonymous online survey likely minimised this bias, as self-administration of questionnaires can increase the willingness to disclose sensitive information as compared to face-to-face or telephone interviews [27]. Furthermore, this study looked at differences in time using the same questionnaires, so this bias is likely negligible.

In conclusion, our study provides the first evidence for the effectiveness of the SUPRANET Community programme on creating attitudinal change in the general public. The results revealed that respondents in the intervention regions considered professional help to be more valuable and were more likely to be familiar with the Dutch helpline than respondents in the control region. In contrast to our hypothesis, we found no evidence for an exposure–response relationship.

## Data Availability

The data that support the findings of this study are available from the corresponding author upon reasonable request.
